# Four‐step approach to efficiently develop capillary gel electrophoresis methods for viral vaccine protein analysis

**DOI:** 10.1002/elps.202000107

**Published:** 2020-07-27

**Authors:** Lars Geurink, Ewoud van Tricht, Justin Dudink, Bojana Pajic, Cari E. Sänger – van de Griend

**Affiliations:** ^1^ Janssen Vaccines and Prevention B.V. Leiden The Netherlands; ^2^ Faculty of Pharmacy Department of Medicinal Chemistry Biomedical Centre Uppsala University Uppsala Sweden; ^3^ Kantisto BV Baarn The Netherlands

**Keywords:** Analytical quality by design, CE‐SDS, Inactivated polio virus, Mini‐hemagglutinin, Viral vaccine protein

## Abstract

Vaccines against infectious diseases are urgently needed. Therefore, modern analytical method development should be as efficient as possible to speed up vaccine development. The objectives of the study were to identify critical method parameters (CMPs) and to establish a set of steps to efficiently develop and validate a CE‐SDS method for vaccine protein analysis based on a commercially available gel buffer. The CMPs were obtained from reviewing the literature and testing the effects of gel buffer dilution. A four‐step approach, including two multivariate DoE (design of experiments) steps, was proposed, based on CMPs and was verified by CE‐SDS method development for: (i) the determination of influenza group 1 mini‐hemagglutinin glycoprotein; and (ii) the determination of polio virus particle proteins from an inactivated polio vaccine (IPV). The CMPs for sample preparation were incubation temperature(s) and time(s), pH, and reagent(s) concentration(s), and the detection wavelength. The effects of gel buffer dilution revealed the CMPs for CE‐SDS separation to be the effective length, the gel buffer concentration, and the capillary temperature. The four‐step approach based on the CMPs was efficient for the development of the two CE methods. A four‐step approach to efficiently develop capillary gel electrophoresis methods for viral vaccine protein analysis was successfully established.

**Abbreviations**
2‐ME: β‐mercapthoethanol
AQbD: analytical quality by design
ATP: analytical target profile
BCA: bicinchoninic acid assay
BFS: bare fused silica
CE‐SDS: capillary electrophoresis sodium dodecyl sulphate
CMP: critical method parameter
DoE: design of experiments
HA: haemagglutinin
HIV: human immunodeficiency virus
IPV: inactivated polio vaccine
NIBSC: National Institute for Biological Standards and Control
PNGase F: N‐glycosidase F
RP‐HPLC: reversed phase high‐performance liquid chromatography
RSV: respiratory syncytial virus
SARS‐CoV‐2: severe acute respiratory syndrome coronavirus 2
UV: ultraviolet
WHO: World health organization

## 1 Introduction

Infectious diseases caused 10.4 million deaths worldwide in 2017, according to Ritchie and Roser [1]. The WHO reckons that vaccination is one of the most effective ways to prevent infectious diseases [2]. Vaccines help the body to recognize pathogens and protect the body against infectious diseases. Nevertheless, vaccines are not available against all infectious diseases, e.g., respiratory syncytial virus (RSV), human immunodeficiency virus (HIV), Severe acute respiratory syndrome‐coronavirus‐2 (SARS‐CoV‐2), Zika virus, or vaccines require improvement to be more effective (e.g., influenza, polio). Therefore, further vaccine development is required.

The development of vaccines is often tight on timelines, hence established techniques are frequently chosen for analysis because methods can be developed quickly. However, these established techniques may lack resolution, throughput, and overall method performance. Implementation of modern techniques requires good fundamental understanding and experience of the technology and its best practice, as well as of the analytes and the application field, and thus decrease method development lead times and improve the overall method performance and robustness.

An example of an application that improved method development lead times and method performance is the automated monoclonal antibody (mAb) analysis by capillary gel electrophoresis (CGE). Commercialized off‐the‐shelf products have been developed to replace the manual SDS‐PAGE [3, 4, 5, 6, 7, 8, 9]. The use of the off‐the‐shelf CE‐SDS products and applications are well established and are, nowadays, often part of the standard mAb product release testing. The CE‐SDS application is also frequently used for non‐mAb proteins such as viral proteins [10, 11, 12, 13, 14, 15, 16, 17, 18, 19]. However, the off‐the‐shelf CE‐SDS products cannot always be applied directly for the analysis of non‐mAb pharmaceutical proteins. Adjustment and optimization of the CE‐SDS application is often required to be able to fulfill pharmaceutical requirements, as captured in the analytical target profile (ATP) [20, 21]. Adjustment and optimization of Off‐the‐shelf applications can be difficult due to the proprietary nature of off‐the‐shelf applications. Thus, this report focuses on identifying the most important (critical) method parameters and proposing an approach to develop and validate a CE‐SDS method for vaccine protein analysis. The proposed approach was subsequently tested on influenza G1 mini‐hemagglutinin protein vaccine (mini‐HA) and inactivated polio vaccine (IPV).

## 2 Materials and methods

### 2.1 Chemicals and materials

Viscosities and conductivities were determined on an AB Sciex CESI 8000 plus with DAD instrument with an Agilent bare fused silica (BFS) capillary 50 μm id, total length 65 cm, and with 2‐phenoxy ethanol as tracer. All CE‐SDS analyses were performed on an Agilent 7100 CE instrument with DAD and an Agilent BFS capillary 50 μm id, total length 33 cm. The SDS‐MW gel buffer, 10 kDa internal standard, IgG control standard, basic wash (0.1 M NaOH), acidic wash (0.1 M HCl), and SDS‐MW size standard as part of the SDS‐MW assay kit and IgG purity heterogeneity assay kit were from AB Sciex. The 10% SDS solution was obtained from Invitrogen, N‐glycosidase F from Roche, and *O*‐glycosidase from New England Boilabs. Triton X‐100, 2‐mercaptoethanol, and neuraminidase from *Arthrobacter ureafaciens* were purchased from Sigma–Aldrich. Influenza antigen standards, purified inactivated viruses, were purchased from NIBSC. Inactivated virus bulk influenza, IPV, and mini‐HA samples were all manufactured at Janssen Vaccines (Leiden, the Netherlands). Instrument control and data analysis software was Agilent ChemStation (B.04.03) for IPV and Waters Empower 3 (Build No. 3471 database version 7.21.00.00, feature release 2) for mini‐HA. Ready‐made bare‐fused silica capillaries of 50 μm id and total length of 33 cm from Agilent Technologies were used. Gel buffer dilutions were made with Milli Q‐water on an analytical balance and sonicated after homogenization.

### 2.2 Viscosity determination

The dynamic viscosity (η) was determined by the application of an hydrodynamic pressure (ΔP) of 100 mbar on one side of the a capillary with a length (L) of 33 cm and an inner diameter (d) of 50 μm, homogeneously filled with the BGE solution and determining the velocity (v_gem_) of a tracer. The velocity of the tracer was determined by measuring the migration time (t_m_) over an effective length of (L_eff_) of 8.5 cm. The dynamic viscosity was thereafter calculated making use of Equation (1), based on Poiseuille's law.

(1) $$\eta =\frac{\Delta P\cdot d^{2}}{32\cdot v_{gem}\cdot L}=\frac{\Delta P\cdot d^{2}}{32\cdot \frac{L_{eff}}{t_{m}}\cdot L}\;$$

### 2.3 Conductivity determination

A 33 cm (total length), 50 μm id BFS capillary was filled with gel buffer. The current was determined at an applied voltage of 10 kV. The conductivity (κ) was calculated based on the capillary length (L), capillary cross‐sectional area (A), the applied voltage (U) and the observed current (I), Equation (2), based on Ohm's and Pouillet's laws.

(2) $$\kappa =\frac{I\cdot L}{U\cdot A}$$

### 2.4 CE‐SDS sample preparation

The IgG control and SDS‐MW size standard were prepared according to the AB Sciex IgG application guide [5]. Protein samples in a specific protein's formulation buffer were reduced at 100°C for 10 min in 1:22%, v/v, SDS solution (10% w/v) and 1:22%, v/v, 2‐mercaptoethanol. Deglycosylation was performed by incubation at 37°C for 3 h in 1:7, v/v, deglycosidase and 1:7, v/v, Triton X‐100. After deglycosylation, 1:15, v/v, SDS solution (10% w/v) was added to the sample.

### 2.5 CE‐SDS analysis

A 33 cm total length (24.5 cm effective length), 50 μm id BFS capillary was preconditioned by flushing with 0.1 M NaOH at 4 bar for 10 min, 0.1 M HCl at 4 bar for 3 min, Milli Q‐water at 4 bar for 2 min, and CE‐SDS gel buffer at 4 bar for 10 min. Samples were injected at 100 mbar for 100 s. Separation was achieved by applying –20 kV with 2 bar pressure applied on both ends of the capillary. The UV‐absorbance signal was recorded at 214 nm.

### 2.6 Protein concentration determination

The Influenza virus concentration was determined by RP‐HPLC according to Kapteyn et al. [22, 23]. Reduced and alkylated samples were injected on a polystyrene POROS^®^ R1/10 (2.1 mm  × 100 mm) column (PerSeptive Biosystems Inc.) with a column temperature of 65°C. The HA protein was eluted with a flow of 0.8 mL/min with an acetonitrile gradient of 20–100% in 6.5 min. TFA was added to the mobile phases in a concentration of 0.098%, v/v. The signal was recorded at UV 214 nm and the HA concentrations were determined against a NIBSC reference standard calibration curve.

The polio vaccine concentration was determined using the BCA protein quantitation assay kit (Pierce^TM^), based on the BCA reaction as described by Smith et al. [24].

The mini‐HA protein concentration was determined by OD280 with a theoretical extinction coefficient of 1.27 m^2^/kg calculated according to Gill et al. [25].

### 2.7 Design of Experiments (DoE)

The experimental design for the optimization experiments, data interpretation, and statistical testing were performed with the “DoE, optimal design” function in JMP V12.0 (SAS) software. Statistical significance tests and prediction profilers available in the JMP fit model platform were used to evaluate the effect of the factors on the responses. Prediction profiles and surface response plots were used to identify the factor settings leading to the most optimal results jointly for all the responses per optimization step.

## 3 Results and discussion

### 3.1 Critical method parameter assessment

The separation mechanism of CE‐SDS is similar to that of SDS‐PAGE [3, 26, 27]. In both techniques, the proteins in a sample are denatured with SDS and get a uniform charge‐over‐size ratio. The sample is loaded onto either a crosslinked polymer slab‐gel or injected into a capillary, that is filled with a linear non‐crosslinked gel buffer. A voltage is applied, and the proteins migrate through the gel. The gel functions as a sieve and the proteins are separated based on their size. This results in an electropherogram with peaks of which the migration time correlates with the protein size and the peak area with the protein concentration.

The CE‐SDS kit was developed for monoclonal antibodies [5, 6] and can also be used to analyze viral proteins in vaccine development samples [12, 20, 28, 29]. Nonetheless, optimization of the different steps in the method (sample preparation, CGE separation and detection) is needed to establish a method that meets the assay requirements as listed in the analytical target profile (ATP) [26]. Each of the different steps in the method contain many parameters, e.g., incubation and separation temperatures, reagent concentrations, etc. To be able to design a method that meets the assay requirements, it is key to understand which are the critical method parameters (CMPs), i.e., the parameters that have most effect on the method performance. The CMPs require optimization and/or will be controlled [30].

The CMPs for sample preparation and detection were thoroughly studied previously [5, 6, 20, 31–34]. Dilution, desalting [5, 6], reduction, alkylation [31, 32] and deglycosylation [20, 33, 34] impact separation and the selectivity of the method. The use and need for sample preparation is dependent on the nature of the sample and the proteins of interest [26, 33], as well as on the purpose of the method. The sample preparation CMPs to optimize the selectivity and sensitivity of the method were the incubation temperature(s) and time(s), pH, and reagent(s) concentration(s) [26]. The sample preparation and detection procedures used in this study were based on the studies performed by Van Tricht et al. [20].

Detection can be performed with UV or LIF. LIF is frequently used to increase the sensitivity of the method and to reduce baseline noise, but requires an additional derivatization step [35, 36, 37]. An additional derivatization step would add another source of variance to the method, therefore we decided to use UV detection. Detection wavelength is an important CMP for sensitivity, and was set to 214 nm.

For the CE‐SDS kit, there has been ample attention to sample preparation, however, there are few papers on the separation optimization of the dextran‐glycerol gel buffer [3, 20, 29, 32, 38, 39, 40]. Therefore, the focus of the first part of this study was to determine the CMPs for the CE‐SDS separation.

The CE‐SDS separation takes place inside a bare fused silica (BFS) capillary, typically 30–33 cm total length. Capillaries with an internal diameter larger than 50 μm were tested and resulted in increased sensitivities. However, use of larger capillary diameters is limited by increased currents resulting in excessive Joule heating. Capillaries longer than 33 cm did not significantly increase the resolution, however, the run time increased substantially. Therefore, a 50 μm BFS capillary of 33 cm total length was used during all our experiments. An effective length of 8.5 cm resulted in similar resolution compared to an effective length of 24.5 cm for the analysis of inactivated virus bulk influenza samples, and the analysis time decreased by a factor 3. Others reported loss of resolutions when using short‐end injection [41]. Therefore, the capillary effective length was added to the set of key steps for fast method development.

#### 3.1.1 CGE separation—gel buffer composition effects

The most used gel buffer in CE‐SDS is a commercialized proprietary gel buffer. The gel buffer composition was studied previously and optimized for the analysis of mAbs [27]. Interestingly, dilution of the gel buffer resulted in better separation, sensitivity, and shorter analysis times for the analysis of hemagglutinin (HA) in influenza virus and virosome [20]. The gel buffer contains dextran, tris, borate, glycerol, SDS, and EDTA, each added for different reasons as mentioned in the patent application [42]. Dextran is a non‐crosslinked linear polymer with low UV‐absorbance that forms an entangled network with dynamic pores for sieving [42]. The gel pore size depends on the polymer concentration and has an impact on the sieving nature of the gel buffer. Gel buffer dilution was expected to increase the gel polymer pore size and to decrease resolution [27, 38, 43], which is contradictory to the influenza protein analysis results [20].

Dilution of the gel buffer decreases the viscosity as well as decreases the ionic strength of the buffer. A lower viscosity increases the hydrodynamically injected sample volume, and increases the conductivity of the buffer. The conductivity of the gel buffer impacts the sample electrokinetic injection and sample stacking. Gel buffer dilution also decreases the ionic strength and thereby the conductivity of the gel buffer, so the ionic strength and the viscosity have opposing effects on the conductivity of the gel buffer. The final result of the dilution depends on the situation at hand. As it was unclear whether diluting the gel buffer ultimately would result in a higher or lower conductivity, this was determined by measuring the conductivities and viscosities of different gel buffer dilutions at different temperatures.

**Figure 1:**
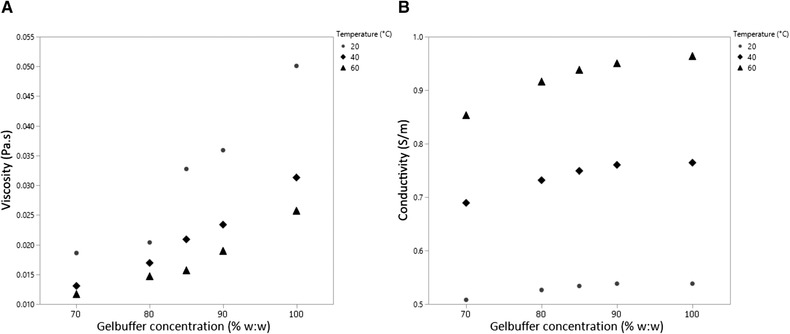
Effects of gel buffer concentration and capillary temperature on (A) the viscosity and (B) the conductivity.

The relative viscosities of 70–100%, w/w, gel buffer concentrations were studied by pushing the gel with 100 mbar pressure over a 50 cm long BFS capillary at temperatures of 20–60°C. A small amount of tracer dissolved in gel buffer was injected to determine the migration time over the effective capillary length and to calculate the viscosity. The decrease in viscosity was non‐linearly related to the gel buffer dilution and the temperature (Fig. 1A). Diluting the gel buffer to 70% decreased the viscosity by a factor 2.7 at 20°C, a factor 2.4 at 40°C, and a factor 2.2 at 60°C.

The effect of the gel buffer dilution on the conductivity was determined by applying 10 kV over a 33 cm long BFS capillary filled with 70–100%, w/w, gel buffer at 20–60°C. Conductivities of 0.96 S/m (60°C), 0.75 S/m (40°C), and 0.22 S/m (20°C) were calculated (Equation (2)) at 100%, w/w, gel buffer (Fig. 1b). Gel buffer dilution from 100% to 70%, w/w, decreased the conductivity with approximately 10%, independent of the temperature. Hence, the ionic strength decrease had a stronger effect on the conductivity than the decrease in viscosity.

**Figure 2:**
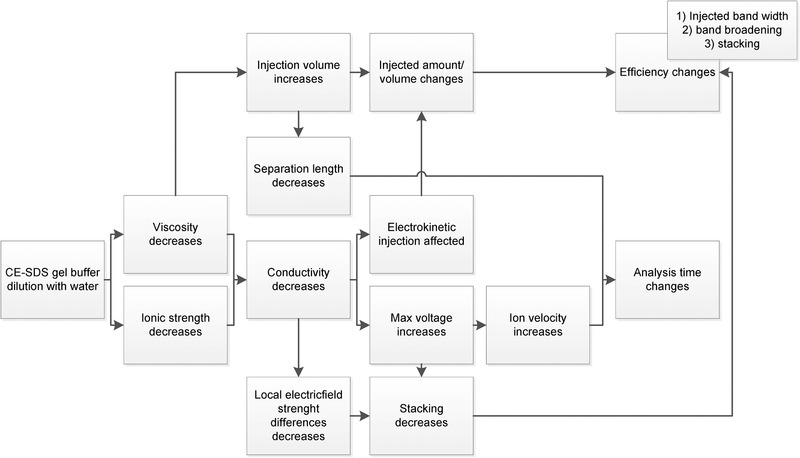
The effects of gel buffer dilution on injection, sample stacking and CGE separation. Dilution of the gel buffer decreases the viscosity as well as decreases the ionic strength of the buffer. A lower viscosity results in an increased conductivity, whereas a lower ionic strength results in a decreased conductivity. In the case of diluting the Sciex CE‐SDS gel buffer with water, the total effect was that the conductivity was decreased (see text). The decreases in viscosity and in conductivity ultimately affect the sample injection, band broadening and sample stacking.

As the conductivity change was dominant, diluting the gel buffer affected electrokinetic injection or hydrodynamic injection differently (Fig. 2). During electrokinetic injection, the amount of protein injected depends on the applied voltage and the relative conductivity of the gel buffer and of the sample. When the gel buffer was diluted and the conductivity decreased, the sample local electric field strength decreased, hence a lower amount of proteins were electrokinetically injected. This was confirmed by injecting inactivated virus bulk influenza samples electrokinetically into different gel buffers with concentrations of 70–90%. At 70%, w/w, gel buffer, the corrected peak areas of the inactivated virus bulk influenza proteins were two times lower compared to 90%, w/w, gel buffer (data not shown).

Generally, we prefer hydrodynamic injection over electrokinetic injection for quantitative analysis [8, 26], as it is generally more precise, non‐selective, and matrix independent, whereas electrokinetic injection is selective and matrix dependent [44, 45]. In hydrodynamic injections, where the injected volume depends on the viscosity and not on the conductivity, gel buffer dilution decreased the viscosity and increased the injected volume, as demonstrated by injecting NIBSC B/Brisbane influenza samples in a capillary filled with gel buffer concentrations between 70% and 100%, w/w. The difference in viscosity at 32.5°C between the 70% and the 100% gel buffer was about 2.6 times. The corrected peak areas of the NIBSC B/Brisbane proteins was 2.6 times higher for a 70% gel buffer concentration than a 100% gel buffer concentration (data not shown).

Although conductivity does not affect hydrodynamic injection, it does affect sample stacking. The conductivity change of diluted gel buffer decreased the conductivity difference with the sample plug and hence reduced stacking capacity, while a larger sample plug was injected. Indeed, the peak efficiency decreased when hydrodynamically injecting NIBSC B/Brisbane influenza samples in 70% gel buffer. The plate numbers reduced in average with a factor 2.4 from 7.3 × 10^4^ to 1.0 × 10^5^ in 100% gel buffer to 1.8 × 10^4^ to 5.0 × 10^4^ in 70% gel buffer. In total, gel buffer dilution increased the hydrodynamically injected plug length, caused broader peaks and lower peak efficiencies, and decreased the stacking capacity. A decrease in efficiency can reduce both the sensitivity and resolution of the method. Naturally, the injection settings could be adjusted to avoid overloading.

**Figure 3:**
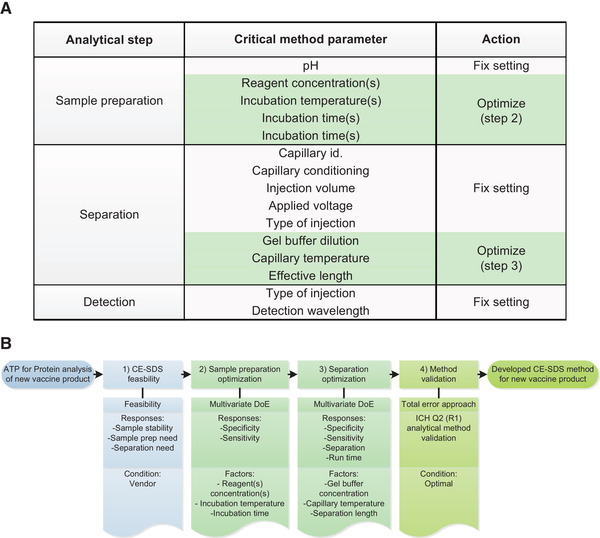
(A) The list of critical method parameters and actions required to develop and validate a CE‐SDS method for vaccine protein analysis. (B) Set of four steps for fast CE‐SDS viral vaccine protein method development and prospective validation.

Figure 2 summarizes the effects of gel buffer dilution of the Sciex CE‐SDS gel buffer with water on the ionic strength, the viscosity, and the conductivity of the gel buffer, and their impact on the analysis time, stacking, separation, and sensitivity. The magnitude of these effects also depends on the capillary temperature. Consequently, the gel buffer dilution, the capillary temperature, and the capillary effective length were considered CMPs that need to be optimized for each analyte and method. An overview of the CMPs and actions required to develop and validate a CE‐SDS method for vaccine protein analysis is depicted in Fig. 3A.

After identification of the CMPs, we defined a set of four steps to quickly develop a CE‐SDS viral protein analysis method (Fig. 3B). First, the feasibility of the default conditions as proposed by SCIEX [5] was assessed and the sample stability and the need for sample preparation and separation optimization evaluated.

The sample preparation was optimized in the second experiment, and the CGE separation was optimized in the third experiment. Both the optimization of the sample preparation and the separation were performed with multivariate DoE approaches. The CMPs to be optimized for sample preparation were the reagent(s) concentration(s), and incubation temperature(s), and incubation time(s). The CMPs to be optimized for separation were the gel buffer concentration, the capillary temperature, and the separation length.

The proposed set of key experiments was applied for the development of CE‐SDS methods for two different viral vaccine protein applications, (i) the determination of influenza group 1 mini‐haemagglutinin mini‐HA(universal influenza vaccine) glycoprotein, and (ii) the determination of polio virus particle proteins from an inactivated polio vaccine (IPV).

### 3.2 Applications

#### 3.2.1 Mini‐hemagglutinin

**Figure 4:**
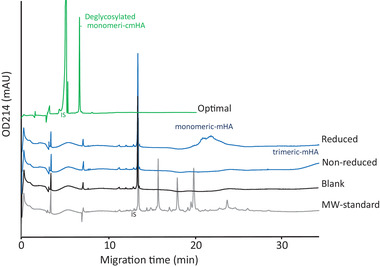
Feasibility and optimization of CE‐SDS for G1 mini‐HA protein purity analysis, with the MW, sizing standard (MW‐standard), mini‐HA formulation buffer (Blank), non‐reduced mini‐HA from the feasibility experiment (Non‐reduced), reduced mini‐HA from the feasibility experiment (Reduced), deglycosylated and reduced mini‐HA at the selected conditions for sample preparation and separation (Optimal). The Internal standard (IS) is 10 KD standard. Separation conditions were 100% gel buffer, 20°C capillary temperature, hydrodynamic injection at 100 mbar for 100 s, and a capillary effective length of 24.5 cm for MW‐standard, Blank, Non‐reduced and Reduced. For the Optimal condition the same conditions applied with a capillary effective length of 8.5 cm instead of 24.5 cm. At the selected conditions, a system peak was observed at 5.0–5.6 min that was separated from the IS at 5.7 min, and the deglycosylated‐monomeric‐mini‐HA peak was observed at 7.2 min.

A Group 1 mini‐HA glycoprotein was developed as part of a universal influenza vaccine [46, 47]. Mini‐HA is a homo‐trimeric protein and has multiple deglycosylation sites with a high degree of variability in the glycosylation composition. The feasibility experiment resulted in a protein peak profile comparable to SDS‐PAGE. The untreated samples resulted, as expected, in broad peaks with migration times of 19–23 min or >30 min (Fig. 4), caused by the highly heterogenous glycosylation of the protein. The deglycosylated samples, non‐reduced or reduced, resulted in single peaks at relative migration times to the 10 kDa marker as expected from the protein theoretical molecular weights and comparable to SDS‐PAGE. The purpose of the method was protein primary structure purity for process development support, hence deglycosylation was included in the sample preparation. Sample preparation including reduction and deglycosylation was optimized to minimize the number of additional peaks caused by inefficient deglycosylation or induced fragmentation, and to minimize the sample preparation duration. Seven continuous‐scale factors, viz. reduction time, reduction temperature, triton concentration, PNGase F concentration, Neuraminidase concentration, O‐glycosidase concentration, and deglycosylation time, were studied each at three levels where the responses of interest were migration time, peak height, resolution, and deglycosylation efficiency. An I‐optimal response surface DoE consisting of a total of 43 experimental runs was used (Supporting Information Table S1). Each experimental run corresponded to a unique combination of the levels of the seven factors and the runs were grouped into three random blocks each executed with a maximum of 15 samples a day.

The factors studied for the separation optimization DoE were gel buffer concentration, capillary temperature, and injection volume, each at three levels, and effective length at two levels. The responses of interest for the separation optimization were peak height, resolution, migration time, and corrected peak area. An I‐optimal response surface DoE consisting of 20 experimental runs was used (Supporting Information Table S2). The 20 runs were grouped into five random blocks where the factor capillary temperature was held constant within a block, because randomization of the capillary temperature would result in time‐out errors of the instrument. Therefore, this DoE is a split‐plot design with the whole plots defined by the levels of the capillary temperature.

The described I‐optimal response surface designs allowed for the estimation of the main effects, quadratic effects, and two‐way interactions between the factors as well as the random block‐to‐block variation per optimization step. Prediction profiles and surface response plots were used to identify the factor settings leading to the most optimal results jointly for all the responses per optimization step. The optimal sample preparation conditions for mini‐HA were 0.45% w/v SDS, 4.5% v/v 2‐ME, and incubation for 10 min at 70°C, followed by 0.66% v/v Triton X‐100, 6.6% v/v of deglycosylation enzymes, and incubation for 1 h at 37°C, and addition of 0.32% w/v SDS and 1.6% v/v 10 kDa internal standard.

Gel buffer dilution only showed limited improvement on the resolution. As it is easier to work with off‐the‐shelf solutions, undiluted gel buffer was selected. An effective length of 8.5 cm was selected since it resulted in a 3 × lower migration time compared to an effective length of 24.5 cm, and did not significantly impact the resolution nor the corrected peak area. The other separation conditions were 20°C capillary temperature, and hydrodynamic injection at 100 mbar for 100 s. With these conditions, a single mini‐HA peak was obtained that was separated from in‐process sample matrix components and mini‐HA degradation products. The method was successfully validated for (i) quantitative purity determination of mini‐HA for process development support, and (ii) quantitative purity measurements of mini‐HA protein for product characterization and stability determination purposes. The corrected peak area repeatability was 0.8% RSD (*n* = 9), in a concentration range of 0.50–3.13 mg/mL and the determined purities were 98–99% (*n* = 27). Because the determined purities determined in the concentration range were between 98 and 99%, the method was linear and accurate. The LOQ for the method was defined as the lowest level of the required range, and as such was 0.50 mg/mL mini‐HA. However, lower concentrations are certainly achievable if required.

#### 3.2.2 Inactivated Polio Vaccine

The IPV consists of inactivated polio virions of three different wild‐type strains, i.e., MEF‐1, Saukett, and Mahony. A polio virion capsid is built of four different proteins and each strain has different VP1, VP2, VP3, and VP4 proteins. A strain‐specific identity method was needed for IPV vaccine development.

**Figure 5:**
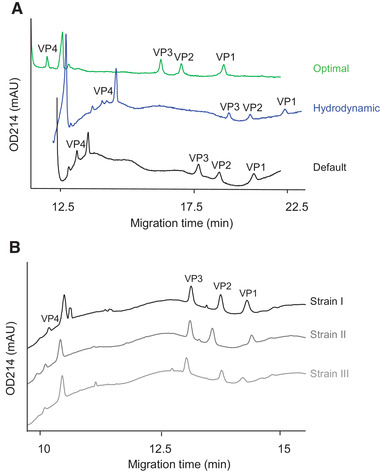
IPV protein (VP1‐4) analysis CE‐SDS method development. (A) Electropherograms of separation optimization results of default conditions with electrokinetic injection (Default), hydrodynamically injected IPV (Hydrodynamic) and the optimal conditions (Optimal), with hydrodynamic injection, 80% gel buffer, and an applied voltage of 20 KV. (B) Electropherograms of three different IPV strains.

Disulfide scrambling in the polio virus proteins can result in tertiary structure heterogeneity and hence in broad peaks. This was confirmed in the feasibility experiment. The the back calculated molecular weights based on the MW‐markerof the individual proteins were as expected and depending on the strain, around 38 kDa (VP1), 31 kDa (VP2), 28 kDa (VP3), and 7 kDa (VP4). The proteins were not well separated with the method as described by SCIEX [5], as peaks were tailing, sample analysis times were long, and the sensitivity was low, indicating the need for protein reduction and separation optimization. The IPV sample preparation optimization experiment included the reduction temperature, the reduction time, the 2‐mercaptoethanol concentration, and the SDS concentration, while the separation optimization experiment comprised the injection volume, the gel buffer concentration, and the effective length. The factors for both experiments were studied with a full‐factorial two‐level design (Supporting Information Tables S3 and S4), so only their main effects on the migration time, corrected peak area, peak height, and resolution were studied. Factor settings that lead to the most optimal results jointly for the responses were identified from the main effects models using the same approach as described for mini‐HA1. The sample preparation optimization experiment resulted in symmetric peaks at denaturation and reduction conditions of 2% v/v SDS and 7% v/v 2‐ME, and 20 min incubation at 100°C. The separation optimization aimed for separation of the 4 viral proteins for each strain, with resolutions >1.7 and peaks detected at a poliovirus concentration of 15 μg/mL. A full factorial separation optimization DoE resulted in the optimal separation conditions, hydrodynamic injection at 100 mbar for 100 s, 80% v/v gel buffer, an applied voltage of –20 kV, an effective length of 24.5 cm, and a cassette temperature of 20°C (Fig. 5A). At these conditions, the four different proteins of each strain (MEF‐1, Saukett, and Mahony) were separated, with resolutions >1.7, and a detection limit at 10 μg IPV/mL (Fig. 5B). The migration time intermediate precision was 0.4–0.8% RSD (*n* = 16). The strains could be identified based on the migration time of the four peaks. Additionally, relative peak area precision for the different proteins was 8–16% RSD (*n* = 8) and the peak areas fitted linearly with the protein concentration (*R* ^2^ > 0.98), indicating that the CE‐SDS method for the analysis of IPV had the potential to be used for quantitative purity as well, although the method was not optimized and validated for this purpose. The complete experimental data set was obtained within 4 days.

## 4 Concluding remarks

The proposed four‐step approach was evaluated by CE‐SDS method development and qualification for IPV proteins and mini‐HA. In both cases, the conditions as proposed by the vendor did not result in a method that met the ATP, the sample preparation and separation required adjustment and optimization. The optimized sample preparation and separation conditions did meet the ATP requirements. The optimal settings for the CMPs differed per analytical target. Important for method optimization are protein‐specific knowledge such as the number of proteins, protein size(s), and the protein heterogeneity such as posttranslational modifications, as well as method‐specific requirements such as the method purpose, selectivity, accuracy, precision, sensitivity, robustness, or short analysis times. The IPV protein analysis method required qualitative analysis of viral capsid proteins in IPV formulation buffer. The mini‐HA CE‐SDS method required quantitative purity determination of a single glycoprotein in process intermediate sample matrices and during stability studies. The same method development approach resulting in significantly different conditions for these two test cases underlines the general applicability of the approach. The four key experiments can be further tailored whenever needed. For example, sample preparation steps such as derivatization for fluorescence detection, could be added. Additionally, electrokinetic injection instead of hydrodynamic injection could be evaluated. A water plug could be implemented to change the local field strength difference and improve stacking [20, 29, 44, 48]. In the presented approach, the gel buffer was diluted with water, thus diluting all components equally. To improve the separation optimization further, rather than maintaining the relative concentrations constant, the concentration of each gel buffer component could be studied. In general, a good understanding of the effects of the CMPs increases the robustness of a method and leads to an efficient approach in method development.

The four steps can be performed within week(s), given the materials are present, instruments available, and operators trained. Altogether, for the presented cases, the total lead time from specifying the need for a vaccine protein analysis method until sample testing with an optimal performing method took considerably shorter than the average lead time for vaccine method development. In the light of the current COVID‐19 outbreak and earlier Zika virus and Ebola virus outbreaks, short analytical quality by design (AQbD) method development lead times contribute to fast responses to reduce the impact of outbreaks.

## Supporting information

Supporting Information
