# Supplementary material for: Four‐step approach to efficiently develop capillary gel electrophoresis methods for viral vaccine protein analysis
Source: Electrophoresis. 2020 Jul 27;42(1-2):10–8. doi: 10.1002/elps.202000107 (PMC7361255; doi:10.1002/elps.202000107)
Supplement: Supplementary file 1 — Supporting Information [file ELPS-42-10-s001.docx]

## Supporting information

Title: Four-step approach to efficiently develop capillary gel electrophoresis methods for viral vaccine protein analysis

Authors: Lars Geurink^a,b^, Ewoud van Tricht^a^, Justin Dudink^a^, Bojana Pajic^a^, Cari E. Sänger – van de Griend^b,c^

Institute:

a Janssen Infectious Diseases and Vaccines B.V., Archimedesweg 4-6, 2333 CN Leiden, The Netherlands

b Uppsala University, Faculty of Pharmacy, Department of Medicinal Chemistry, Biomedical Centre, PO Box 574, SE-751 23 Uppsala, Sweden

c Kantisto BV, Callenburglaan 22, 3742 MV Baarn, The Netherlands

Contact information:

Lars Geurink, MSc.

Archimedesweg 4-6, 2333 CN Leiden, The Netherlands

[lgeurin1@its.jnj.com](mailto:lgeurin1@its.jnj.com)

+31 71 519 7686

Content:

Experimental design tables, with run order and experimental conditions, to optimize I) a CE-SDS method for mini-heamagglutinin (mini-HA) protein primary structure purity for process development support, and II) a CE-SDS method for strain-specific identity determination of inactivated polio virus (IPV) vaccine development.

- Table 1 mini-HA sample preparation optimization experimental design
- Table 2 mini-HA separation optimization experimental design
- Table 3 IPV sample preparation optimization experimental design
- Table 4 IPV separation optimization experimental design
- ATP mini-HA
- ATP IPV

#### Table 1 mini-HA sample preparation optimization experimental design

| Run order | Block | Reduction time (min) | Reduction temperature (°C) | Triton X-100 (µL) | PNGaseF  (µL) | Sialidase  (µL) | O-glycosydase  (µL) | Deglycosylation time  (h) |
| --- | --- | --- | --- | --- | --- | --- | --- | --- |
|  |  | (5; 10; 15) | (60; 70; 80) | (2; 5; 8) | (2; 5; 8) | (2; 5; 8) | (2; 5; 8) | (1; 3.5; 6) |
| 1 | 1 | 5 | 60 | 2 | 5 | 2 | 5 | 3.5 |
| 2 | 1 | 10 | 70 | 2 | 5 | 2 | 2 | 1 |
| 3 | 1 | 15 | 60 | 8 | 2 | 2 | 8 | 6 |
| 4 | 1 | 15 | 60 | 2 | 2 | 2 | 8 | 1 |
| 5 | 1 | 5 | 60 | 8 | 5 | 5 | 8 | 1 |
| 6 | 1 | 5 | 80 | 2 | 2 | 8 | 8 | 3.5 |
| 7 | 1 | 5 | 60 | 2 | 2 | 5 | 2 | 6 |
| 8 | 1 | 15 | 70 | 2 | 2 | 8 | 8 | 6 |
| 9 | 1 | 15 | 70 | 5 | 5 | 5 | 5 | 1 |
| 10 | 1 | 15 | 80 | 8 | 2 | 8 | 2 | 6 |
| 11 | 1 | 5 | 80 | 2 | 8 | 2 | 8 | 6 |
| 12 | 1 | 15 | 60 | 2 | 8 | 8 | 5 | 3.5 |
| 13 | 1 | 5 | 60 | 5 | 8 | 5 | 2 | 3.5 |
| 14 | 1 | 5 | 80 | 8 | 8 | 2 | 2 | 1 |
| 15 | 1 | 10 | 70 | 5 | 2 | 5 | 5 | 3.5 |
| 16 | 2 | 15 | 60 | 2 | 8 | 2 | 2 | 6 |
| 17 | 2 | 10 | 80 | 2 | 2 | 2 | 5 | 6 |
| 18 | 2 | 15 | 60 | 8 | 5 | 8 | 2 | 6 |
| 19 | 2 | 15 | 70 | 8 | 8 | 8 | 2 | 1 |
| 20 | 2 | 5 | 60 | 8 | 2 | 8 | 5 | 6 |
| 21 | 2 | 15 | 80 | 8 | 8 | 5 | 8 | 6 |
| 22 | 2 | 10 | 80 | 2 | 8 | 8 | 2 | 6 |
| 23 | 2 | 10 | 60 | 2 | 2 | 8 | 2 | 1 |
| 24 | 2 | 10 | 60 | 5 | 5 | 5 | 8 | 6 |
| 25 | 2 | 5 | 70 | 2 | 8 | 5 | 5 | 1 |
| 26 | 2 | 5 | 70 | 8 | 8 | 8 | 8 | 3.5 |
| 27 | 2 | 5 | 80 | 8 | 2 | 8 | 2 | 1 |
| 28 | 2 | 15 | 80 | 8 | 5 | 2 | 8 | 1 |
| 29 | 2 | 10 | 70 | 5 | 5 | 5 | 5 | 3.5 |
| 30 | 2 | 5 | 70 | 8 | 5 | 2 | 2 | 6 |
| 31 | 3 | 5 | 70 | 5 | 2 | 2 | 8 | 1 |
| 32 | 3 | 15 | 60 | 8 | 2 | 2 | 2 | 1 |
| 33 | 3 | 15 | 80 | 2 | 2 | 5 | 2 | 1 |
| 34 | 3 | 10 | 60 | 8 | 8 | 2 | 5 | 6 |
| 35 | 3 | 10 | 70 | 5 | 5 | 5 | 5 | 3.5 |
| 36 | 3 | 5 | 80 | 8 | 2 | 5 | 8 | 6 |
| 37 | 3 | 15 | 60 | 8 | 2 | 8 | 8 | 1 |
| 38 | 3 | 5 | 60 | 2 | 8 | 8 | 8 | 6 |
| 39 | 3 | 5 | 80 | 5 | 5 | 8 | 5 | 6 |
| 40 | 3 | 10 | 70 | 8 | 5 | 5 | 2 | 3.5 |
| 41 | 3 | 10 | 80 | 5 | 8 | 8 | 8 | 1 |
| 42 | 3 | 15 | 80 | 5 | 5 | 2 | 2 | 3.5 |
| 43 | 3 | 15 | 60 | 5 | 8 | 2 | 8 | 1 |

#### Table 2 mini-HA separation optimization experimental design

| Run order | Block | Gel buffer concentration (%) | Capillary temperature (°C) | Injection Volume  (mbar.s) | Effective length  (cm) |
| --- | --- | --- | --- | --- | --- |
|  |  | (70; 85; 100) | (20; 25; 30) | (5000; 7500; 10000) | (8.5; 24.5) |
| 1 | 1 | 70 | 25 | 5000 | 24.5 |
| 2 | 1 | 85 | 25 | 7500 | 8.5 |
| 3 | 1 | 100 | 25 | 10000 | 24.5 |
| 4 | 1 | 85 | 25 | 7500 | 8.5 |
| 5 | 2 | 85 | 30 | 7500 | 8.5 |
| 6 | 2 | 100 | 30 | 7500 | 24.5 |
| 7 | 2 | 85 | 30 | 10000 | 24.5 |
| 8 | 2 | 70 | 30 | 5000 | 8.5 |
| 9 | 3 | 100 | 30 | 5000 | 24.5 |
| 10 | 3 | 85 | 30 | 5000 | 24.5 |
| 11 | 3 | 100 | 30 | 10000 | 8.5 |
| 12 | 3 | 70 | 30 | 7500 | 24.5 |
| 13 | 4 | 100 | 20 | 5000 | 24.5 |
| 14 | 4 | 70 | 20 | 10000 | 24.5 |
| 15 | 4 | 70 | 20 | 5000 | 8.5 |
| 16 | 4 | 100 | 20 | 10000 | 8.5 |
| 17 | 5 | 85 | 25 | 7500 | 24.5 |
| 18 | 5 | 100 | 25 | 5000 | 8.5 |
| 19 | 5 | 70 | 25 | 10000 | 8.5 |
| 20 | 5 | 85 | 25 | 7500 | 24.5 |

#### Table 3 IPV sample preparation optimization experimental design

| Run order | Reduction temperature (°C) | Reduction time (min) | β-mercaptoethanol concentration (% v:v) | SDS concentration (% w:v) |
| --- | --- | --- | --- | --- |
|  | (80; 100) | (10; 20) | (7; 14) | (0.1; 0.2) |
| 1 | 80 | 10 | 7 | 0.1 |
| 2 | 80 | 10 | 7 | 0.2 |
| 3 | 80 | 10 | 14 | 0.1 |
| 4 | 80 | 10 | 14 | 0.2 |
| 5 | 80 | 20 | 7 | 0.1 |
| 6 | 80 | 20 | 7 | 0.2 |
| 7 | 80 | 20 | 14 | 0.1 |
| 8 | 80 | 20 | 14 | 0.2 |
| 9 | 100 | 10 | 7 | 0.1 |
| 10 | 100 | 10 | 7 | 0.2 |
| 11 | 100 | 10 | 14 | 0.1 |
| 12 | 100 | 10 | 14 | 0.2 |
| 13 | 100 | 20 | 7 | 0.1 |
| 14 | 100 | 20 | 7 | 0.2 |
| 15 | 100 | 20 | 14 | 0.1 |
| 16 | 100 | 20 | 14 | 0.2 |

#### Table 4 IPV separation optimization experimental design

| Run order | Injection volume  (mbar . s) | Gel buffer concentration (%) | Effective length (cm) |
| --- | --- | --- | --- |
|  | (5000; 7500; 10000) | (80; 100) | (8.5; 24.5) |
| 1 | 10000 | 100 | 24.5 |
| 2 | 10000 | 100 | 24.5 |
| 3 | 10000 | 100 | 24.5 |
| 4 | 10000 | 100 | 24.5 |
| 5 | 7500 | 100 | 24.5 |
| 6 | 5000 | 100 | 24.5 |
| 7 | 10000 | 100 | 8.5 |
| 8 | 7500 | 100 | 8.5 |
| 9 | 5000 | 100 | 8.5 |
| 10 | 10000 | 100 | 24.5 |
| 11 | 10000 | 80 | 24.5 |
| 12 | 7500 | 80 | 24.5 |
| 13 | 5000 | 80 | 24.5 |
| 14 | 10000 | 80 | 8.5 |
| 15 | 7500 | 80 | 8.5 |
| 16 | 5000 | 80 | 8.5 |
| 17 | 10000 | 100 | 24.5 |

#### ATP mini-HA

| **Method characteristic** | **Target** |
| --- | --- |
| Purpose | Protein purity for process development support (early phase development) |
| Specificity | Monomeric Mini-HA, mini-HA degradants, host cell proteins in process intermediate sample matrices |
| LOQ | ≤ 0.5 mg/mL |
| Precision   - Repeatability (within run) - Intermediate precision | ≤ 2% RSD  N.D. (Sample comparison only within the same run, not between runs) |
| Accuracy / Linearity | Consistent % corrected peak areas over the tested range confirms accuracy and linearity |
| Stability indicating power | Decrease in corrected peak area of mini-HA and increase of impurities upon stressing the reference sample. |

#### ATP IPV

| **Method characteristic** | **Target** |
| --- | --- |
| Purpose | IPV Strain identity of purified sample for early phase development |
| Specificity | VP1-4 specific fingerprint per IPV strain in formulation buffer |
| LOD | ≤ 50 µg/mL |
| Precision   - Repeatability* - Intermediate precision (migration time)† - Intermediate precision (Corrected peak area)* | ≤ 10% RSD  ≤ 2% RSD  N.D. (Sample comparison only within the same run, not between runs) |
| Accuracy / Linearity* | Consistent % corrected peak areas over the tested range confirms accuracy and linearity |

* Not relevant for this identity method, in scope for future quantitative purity method

† Intermediate precision with different reagent lots and at different days
